# Prolonged Fasting as a Cause of Deep Vein Thrombosis: A Case Report

**DOI:** 10.1055/s-0043-57225

**Published:** 2023-04-20

**Authors:** Guillaume Roberge, Carine Samson, Grégoire Le Gal, Anthony Calabrino

**Affiliations:** 1 Centre d'Excellence des Maladies Vasculaires, Centre Hospitalier Universitaire de Québec, Université Laval, Hôpital Saint-François d'Assise, Québec, Canada; 2 MaClinique Médicale Lebourgneuf, Québec, Canada; 3 Department of Medicine, Ottawa Hospital Research Institute, University of Ottawa, Ottawa, Ontario, Canada

**Keywords:** fasting, thrombosis, venous thrombosis, dehydration, anticoagulation

## Abstract

**Background** Intermittent fasting is becoming more popular as health benefits are described in recent literature. Various forms of fasting exist, one of them involving a zero-calorie diet and drinking only water. However, the safety of water-only fasting is still not well established. We report a case of a man who developed a lower limb deep vein thrombosis at the end of a 2-week water-only fasting and characterized by an initial period of 5 days of no food and no water intake. We reviewed literature regarding potential links between fasting and venous thromboembolism (VTE).

**Clinical Approach** We believe that fasting can induce important dehydration, leading to hypercoagulability and then contribute to the development of a venous thrombosis. The patient was treated with apixaban for 3 months as is recommended in patients with a provoked event caused by a transient risk factor. No thrombotic recurrence was observed during the 6-month follow-up.

**Conclusion** The public needs to be aware of the potential life-threatening complications associated with important dehydration in the setting of medically unsupervised fasting, and these might include VTE. Whether a VTE with dehydration as the only identified risk factor should be approached as a low recurrence risk situation or not still needs to be clarified.

## Introduction

Intermittent fasting is becoming more popular as health benefits are described in recent literature. Weight loss, reduction in abdominal fat, decrease inflammation, lowering blood pressure, and reduction of insulin resistance are some of the observed effects of dietary restriction. 1 Various forms of fasting exist but medical supervision is usually recommended, especially when food restriction is more important or for a prolonged period of time. 2 3 4

Water-only fasting is defined by restriction of every type of food (zero-calorie diet) except water. In the 1960s and 1970s, fasting duration of 60 days and more was considered for important weight loss, but this practice has been abandoned due to important complications and deaths. At that time, fasts were improperly done due to lack of physicians' and researchers' knowledge about safety and risks. 5 This kind of fast has also been described in nonobese persons for religious purposes. 6 7

Nevertheless, potential health benefits are still reported after shorter course of water-only fasting done under appropriate medical supervision. 4 8 9 However, safety of such food restriction is still not well established and relies on limited data. 4 5

We describe the case of a man who developed a right lower limb proximal deep vein thrombosis (DVT) in the context of unsupervised water-only fasting.

## Case Presentation

A 59-year-old male, with a body weight of 70 kg and body mass index of 24 kg/m ^2^ , presented with a progressive right lower limb pain and edema starting at the end of a voluntary 2-week water-only fasting period. He was not known for any relevant past medical or family history. In the last year, the patient had undergone three medically unsupervised water-only fasting periods of 1 week for potential health benefits purposes. He had progressively increased not only the frequency of these fasts but also their duration up to 2 weeks. In his second 2-week fasting, the patient was still walking 5 km per day, lost around 10 kg (14% of total body weight), and started drinking 500 mL of water per day only on day 6 (i.e., no water for the first 5 days). He did not take any food supplements or weight loss drugs. The last 3 days of the fast (days 12 to 14), the patient reduced his activities and his walks because of fatigue but he was not bedridden. While he was resuming food intake (only fruit juice), he developed his right leg symptoms. Ten days after, he sought medical attention, and a diagnosis of right lower limb proximal DVT (peroneal veins up to the common femoral vein) was made. There were no identified risk factors for venous thromboembolism (VTE) (no recent surgery, trauma or admission to hospital, no long travel, no family history of VTE, no known comorbidities, no obesity, no active smoking, no varicose veins, no recent coronavirus disease infection) and blood work was normal at diagnosis. Thrombophilia testing was not done. Apixaban was initiated and the patient's symptoms progressively improved over the next weeks. At 3 months, anticoagulation was discontinued, and a repeated venous ultrasound revealed a residual thrombus without recanalization in the peroneal and femoral veins, but popliteal vein was partially recanalized.

After 6 months of follow-up, he was still presenting slight right leg edema, heaviness while walking, and new varicose veins, but did not have a venous thrombosis recurrence.

## Discussion

### Fasting Causes Dehydration

Complex physiologic processes occurring with fasting have been hypothetically suggested as a cause of observed increased diuresis and potential resulting dehydration. 10 The decrease of glucose intake impairs sodium reabsorption as glucose-sodium cotransport in renal proximal tubules is inhibited. This ensued natriuresis is not compensated enough by the distal tubular sodium reuptake and then explains resulting osmotic diuresis and subsequent dehydration. 10 11 12

As the patient was taking no calorie and 500 mL of water per day starting only on day 6, and experiencing a 10-kg weight loss in 14 days (weight that he regained in 1 week after restarting food intake), significant dehydration was most likely present. Also, it is improbable that only the first 5 days with no intake (no food, no water) explain the whole dehydration as weight loss was progressive over the 14 days. Despite water intake, raised hematocrit and blood urea nitrogen were reported after water-only fasting in two cases, reflecting potential dehydration. 7 13

### Dehydration is a Risk Factor of VTE

Dehydration is one of the potential explanations put forward to explain the increased VTE incidence observed after prolonged travel. 14 This state likely results in hypercoagulability induced by hemoconcentration and hyperviscosity as reflected in part by increased biochemical parameters such as hematocrit, plasma proteins, plasma, and urine osmolality. 15 Other conditions in which VTE likely occurs because of dehydration have been described such as following ischemic strokes, intense exercises, and gastroenteritis. 16 17 18 19 20 Also, according to some authors, seasonal variation of VTE incidence could hypothetically be related to dehydration, occurring more often with higher temperature. 21

Some literature suggests that theoretically, medically supervised water-only fasting could in fact decrease thrombosis risk. Decreased platelet formation and activity was observed, but this was tested only after 7 days of fasting. 9 Also, increased fibrinolytic activity was described in 12 healthy men after a 60-hour fast in which only water was allowed. 22 However, vascular clinical outcomes under these circumstances were not reported. Of note, these studies do not apply to our unsupervised patient after a 2-week fast.

Important dehydration in this healthy and active patient most likely led to a state of hypercoagulability. It is unknown if an undiagnosed underlying predisposition to thrombosis such as a hereditary thrombophilia was present or if fasting can affect coagulability in another way. We believe that important dehydration in the context of fasting can represent on its own a transient risk factor for VTE. However, it is unknown if it should be interpreted as a major or minor one and deemed at low risk of recurrence. 23 We have not found any previous report of thrombosis complications following fasting. 5 6

Immobility could also represent a risk factor in fasting if the patient becomes confined to bed because of weakness. However, we highly doubt it explains the VTE in our case. Indeed, despite the reported reduction in his activities in the last 3 days of his fast, the patient was still mobile.

A concerted decision was made to treat 3 months with anticoagulation, as recommended with provoked VTE caused by a transient risk factor. 24 No recurrence was observed up to 3 months after stopping the treatment.

There are limitations to the present case, as exact hydration status was not established at the time the DVT occurred considering the patient had regained his usual weight at diagnosis. Also, we do not have any available blood test results at the end of the 2-week fast in correlation with dehydration. This is only one case and firm conclusions cannot be drawn.

## Conclusion

While literature suggests potential benefits from intermittent fasting, the public needs to be aware of the potential life-threatening complications associated with important dehydration in the setting of medically unsupervised fasting, and these might include VTE. Whether VTE caused in the context of dehydration as the only identified risk factor should be approached as a low recurrence risk situation or not still needs to be clarified.

## Highlights

- Intermittent fasting is getting more popular.
- Medically unsupervised fasting can lead to significant dehydration.
- Dehydration is a risk factor for venous thromboembolism.
- Risk of thrombosis recurrence and best management in this setting is uncertain.
